# Associative Cognitive Factors of Math Problems in Students Diagnosed With Developmental Dyscalculia

**DOI:** 10.3389/fpsyg.2018.01907

**Published:** 2018-10-09

**Authors:** Johannes Erik Harold Van Luit, Sylke Wilhelmina Maria Toll

**Affiliations:** Department of Pedagogics and Education, Faculty of Social Sciences, Utrecht University, Utrecht, Netherlands

**Keywords:** dyscalculia, planning, naming speed, memory, attention, diagnosis, protocol

## Abstract

The Dutch protocol, ‘Dyscalculia: Diagnostics for Behavioral Professionals’ (DDBP protocol; Van Luit et al., 2014), describes how behavioral experts can examine whether a student has developmental dyscalculia (DD), based on three criteria: severity, discrepancy, and resistance. In addition to distinguishing the criteria necessary for diagnosis, the protocol provides guidance on formulating hypotheses by describing and operationalising four possible associative cognitive factors of math problems: *planning skills, naming speed, short-term and/or working memory*, and *attention.* The current exploratory and descriptive research aims to describe the frequency of these four primary associative cognitive factors in students with DD from the Netherlands. Descriptive data from 84 students aged 8–18 years showed that deficits in *naming speed* (in particular, in naming numbers) were the most frequent explanation of math problems in children with DD, followed by deficits in *short-term/working memory* and *planning skills*. Deficits in *attention* were the least frequent. The findings are explained in light of current literature, and suggestions for follow-up research are presented.

## Introduction

Many students in primary and secondary education experience problems with mathematics (Geary, 2004). Math problems can have major consequences for their further educational career and for their ability to live independently in society (Every Child a Chance Trust, 2009). Math problems that are extensive and persistent in nature may indicate developmental dyscalculia (DD). Although there is inconsistent use of terminology in the literature, researchers agree that DD refers to the existence of a severe disability in learning mathematics. Ruijssenaars et al. (2016, p. 28) defined DD as a disorder characterized by persistent problems with learning and fluency and/or accurate recall and/or application of mathematical knowledge (facts and understanding). The prevalence of DD is estimated to be between 2 and 3% in students in the Netherlands (Ruijssenaars et al., 2016). Percentages are higher in international research (3–8%), depending on how researchers define such mathematical disorders (Desoete et al., 2004; Dowker, 2005; Shalev et al., 2005). The disability can be highly selective, affecting learners with normal intelligence (e.g., Landerl et al., 2004), although it also co-occurs with other developmental disorders, including reading disorders (Ackerman and Dykman, 1995; Light and DeFries, 1995; Gross-Tsur et al., 1996) and attention deficit hyperactivity disorder (ADHD; Monuteaux et al., 2005).

Within the revised fourth edition of the Diagnostic and statistical manual of mental disorders (DSM-IV-TR; American Psychiatric Association, 2000) the now-obsolete diagnostic criteria for Mathematics Disorder (code: 315.1) were: (A) Mathematical ability, as measured by individually administered standardized tests, is substantially below that expected given the person’s chronological age, measured intelligence, and age-appropriate education; (B) The disturbance in Criterion A significantly interferes with academic achievement or activities of daily living that require mathematical ability; and (C) If a sensory deficit is present, the difficulties in mathematical ability are in excess of those usually associated with it. The fifth edition of the Diagnostic and Statistical Manual of Mental Disorders (DSM-5; American Psychiatric Association, 2013) takes a different approach to learning disorders than previous editions of the manual by broadening the category, in order to increase diagnostic accuracy and effectively target care. Specific learning disorder is now a single, overall diagnosis, incorporating various deficits that impact academic achievement. The criteria describe shortcomings in general academic skills, providing detailed specifiers for the areas of reading, mathematics, and written expression. Diagnosis of this disorder requires persistent difficulties in reading, writing, arithmetic, or mathematical reasoning skills during the formal years of schooling. Symptoms may include inaccurate or slow and effortful reading, poor written expression that lacks clarity, difficulties remembering number facts, or inaccurate mathematical reasoning. Current academic skills must be well below the average range of scores in culturally and linguistically appropriate tests of reading, writing, or mathematics. The individual’s difficulties must not be better explained by developmental, neurological, sensory (vision or hearing), or motor disorders and must significantly interfere with academic achievement, occupational performance, or activities of daily living.

Despite the changes from DSM-IV-TR to DSM-5, it remains necessary to perform extensive diagnostic testing to establish whether DD is present. The Dutch ‘Protocol dyscalculia: Diagnostics for behavioral professionals’ (DDBP protocol; Van Luit et al., 2014) describes how behavioral experts can examine whether a student, from 8 years of age and older, has DD. The DDBP protocol contains guidelines and suggestions about the variables that can be investigated, and the methods used, during a diagnostically examination of DD. Due to its structured and comprehensive nature, the DDBP protocol has now been systematically implemented in many social care settings in education in the Netherlands and Flanders (Belgium). The DDBP protocol deals with the criteria that must be met in order to diagnose DD (Van Luit, 2012; Van Luit et al., 2014), namely:

(1) *The criterion of severity*: there is a significant delay in automated math skills as compared to peers and/or fellow children and a significant delay in mastering the substantive math skills of the various domains. At the end of primary school, for example in sixth grade, there must be a delay of at least 2 years on a standard (national) math test. For such a test this would mean that a student at the end of sixth grade would fail a test designed for children at the end of fourth grade. In earlier grades, for example halfway through fifth grade, this would mean that the student would fail the test designed for students at the end of third grade. At the beginning of fourth grade, a student would fail the test designed for children at the end of second grade. Dyscalculia is rarely diagnosed before the end of third grade.

(2) *The criterion of discrepancy*: there is a significant delay in mathematics with respect to what can be expected of the individual, based on their individual development. In determining dyscalculia, the presence of an average intelligence is not typically required. The cognitive level is mostly assessed by an intelligence test. Children with dyscalculia can have an under- or above-average intelligence level. It is not possible to determine dyscalculia when the student has an intelligence score of 70 or below, because in that case the low mathematical skills are expected relative to the child’s personal abilities. When the total IQ score is between 71 and 85, diagnosing dyscalculia must be done with caution. Mathematics requires a complex skill set that relies on higher cognitive functions. Therefore, it is not realistic to expect that children with an IQ at this level will develop and achieve the same math abilities as their peers with an average IQ score. For these children the lag in mathematical skills needs to be larger (at the end of grade six, at least three years) than the lag of mathematical skills of a person with an average intelligence score (at the end of grade six, at least 2 years).

(3) *The criterion of didactic resistance*: there is a persistent mathematical problem, which is resistant to specialized help. To determine the persistence of the deficit, the structural and specialized help a student had received in mathematics is investigated. Receiving most attention here are past reports of offered help. According to the model of ‘response-to-instruction,’ didactic resistance can only be determined with full certainty when the conditions for all three criteria have been met (Fuchs and Vaughn, 2012). Thus dyscalculia cannot be diagnosed if the third criterion has not been complied with, a condition that also applies to children in secondary school.

Since recent research has increasingly recognized the heterogeneity of DD by differentiating among underlying cognitive deficits (Murphy et al., 2007; Rubinsten and Henik, 2009; Geary, 2011; Kaufmann et al., 2013; Skagerlund and Träff, 2016), identification of DD does not on its own provide sufficient information about the educational needs of an individual student with math problems. The DDBP protocol therefore provides, in addition to the above three criteria, guidance on performing diagnostic research by describing and operationalising four possible associative, or primary, factors related to a student’s math problems: *planning skills, naming speed, short-term and working memory*, and *attention*. (Number sense is also mentioned within the DDBP protocol, but was not taken into account in this research due to issues relating to the time frame.) The five factors (including number sense) are in line with international research on the underlying neurocognitive correlational and causal factors in mathematical difficulties (Träff et al., 2017). Where the diagnosis of DD provides some information about the presence of the problem, identification of these additional factors enables a more complete and integrated picture of an individual student’s educational needs – and thus a sounder basis for appropriate interventions. This might include compensation, remediation and/or dispensation, depending on discovered associative factor(s).

A distinction is made here between primary associative and secondary associative factors. Secondary factors mentioned in the protocol, for example, are work attitude and motivation, self-concept, anxiety, reading problems and delayed or disturbed social-emotional development (e.g., Carey et al., 2016; Sorvo et al., 2017). As mentioned, the DDBP protocol names five primary associative factors; however, these possible factors are certainly not exhaustive. The first primary factor is *planning skills*. Planning processes are required during math tasks for choosing and applying strategies, monitoring calculation(s), applying mathematical knowledge, and checking the answer (Das and Naglieri, 1997). Deficits in planning skills therefore seem to explain an important part of why students with DD have difficulty performing mathematical procedures. Students with DD have been found to have deficits in planning, as compared to students without DD (Kroesbergen et al., 2003). The second primary factor is *naming speed*. Naming speed is the speed of access to (specific) information in long-term memory. A deficit in naming speed can mean that more time and effort is needed during math tasks to make relevant information readily available for solving a task. In students with DD, there is evidence of a deficit in the naming speed of numbers or, alternatively, general deficits in naming speed (D’Amico and Passolunghi, 2009; Mazzocco and Grimm, 2013; Koponen et al., 2017). The third primary factor is *short term and/or working* memory. During math tasks, a large amount of information must be retained and processed. This requires the application of both short-term and working memory. Various studies have found that difficulties in storing, editing, and reproducing auditory information in verbal memory (Berg, 2008) as well as visuospatial information in visual memory (D’Amico and Guarnera, 2005) can underly deficits in DD (Raghubar et al., 2010; Toll et al., 2011). The fourth primary factor is *attention*. Being able to focus and maintain attention ensures that math tasks are accurately represented during problem solving and, further, that math facts are readily and accurately recalled from memory (Passolunghi and Cornoldi, 2000). Also, by maintaining attention, a student can focus on math problems for longer periods of time (Roeyers and Baeyens, 2016). In the case of students with DD, attentional skills are often weaker (Kroesbergen et al., 2003); these students also can have difficulty suppressing responses (i.e., lower inhibitory control; D’Amico and Passolunghi, 2009; Ashkenazi and Henik, 2010; Navarro et al., 2011). The fifth primary factor is *number sense*. Recent research (De Smedt et al., 2009; Fuchs et al., 2010; LeFevre et al., 2010; Kolkman et al., 2013; Schneider et al., 2017) shows that number sense, the ability to process, understand and estimate numerical quantities (Dehaene, 1992), is a predictive factor in the development of math skills. Deficits in number sense appear to be a possible explanation for serious math problems (Mussolin et al., 2010; Piazza et al., 2010; Mazzocco et al., 2011). Research in developmental neuroscience (e.g., Olsson et al., 2016) has even identified neural markers of impairments in numerosity processing in DD (for a review, see Butterworth et al., 2011).

Although recent research has repeatedly linked the five factors to the presence of math problems, few studies (e.g., Navarro et al., 2011) have tested all the factors together. The literature is also missing clinically oriented research involving an adequate number of students diagnosed with persistent mathematical learning disabilities (i.e., DD). The limited amount of research into DD is largely due to issues of feasibility and generalisability. However, research into a target clinical group, whether descriptive or not, can provide valuable information about the presence of the factors in students with DD. Of particular benefit can be information about the frequency with which those students fail to perform, in comparison with their peers.

The current research aimed to describe the frequency of four of the five primary associative factors in students with DD. Otherwise put, it examined in how many children the four factors^1^ could be identified as underlying mechanisms of their math issues. The central research question was, ‘What is the frequency of deficits in planning skills, naming speed, short-term and/or working memory, and attention in children with DD?’ The research objective was twofold. Firstly, the study aimed to examine, per factor, the percentage of students showing deficits in that particular domain. Secondly, it sought to investigate the multifactorial distribution of deficits in these primary factors, as may contribute to DD, i.e., how many students with DD have deficits on one or more of the primary associative factors? Although insight into a limited group of children does not provide information about the strength of the relationship between an associative factor and the presence of math problems per se, it lends support for the outlined diagnostic framework in the DDBP protocol. This may be especially so because of the clinical sample itself and the extensive diagnostic evaluations performed prior to diagnosing DD – and thus prior to inclusion in this research. Empirical evidence regarding these underlying factors can provide additional insight into the nature of such mathematical deficits. As such, it can contribute to accountability of procedures within diagnostic care. This can help clinicians and teachers alike to identify targets of intervention and, as well, enable students with DD to overcome their deficiencies in the field of mathematics.

## Materials and Methods

### Participants

The participants included in this study were 84 students from the Netherlands (8–18 years of age) were visiting a university institute for learning difficulties because of their problems with mathematics and were not diagnosed with DD before. Only those who were diagnosed with DD were included in this study. Diagnostic examination into math difficulties was conducted during the period 2009–2015, and was based on diagnostic research using the three criteria of the DDBP protocol. A consent statement for participation in scientific research was signed by parent(s) or those with parental authority for all participants. **Table 1** describes the 84 students included in the current research. More than three quarters of the students (*n* = 64, 76.2%) were girls. This skewed distribution of sex was remarkable because previous research (Devine et al., 2013) did not reveal such gender differences. The mean intelligence score of the participants was 91.28 (*SD* = 11.30). For almost all students (*n* = 81), the Speed Number Facts Test (SNFT; De Vos, 1992, 2010) had been administered as one component of the diagnostic procedure. The SNFT was used to measure the amount of memorized mathematical facts. The raw average total score on the SNFT was 73.00 (*SD* = 30.83, *n* = 58) on the 2010 version and 67.00 (*SD* = 20.63, *n* = 20) on the 1992 version. In the case of three students, due to their young age and in accordance with the manual, only the addition and subtraction parts were administered. Their average raw score on the 2010 version was 27.50 (*SD* = 0.71, *n* = 2), and on the 1992 version was 19.00 (*n* = 1).

**Table 1:** Participant data for total group by education level.

	*n* (%)	Sex		Age in months			
		
		Boys (%)	Girls (%)	*M*	*SD*	Min	Max
Primary education	56 (66.7)	14 (25.0)	42 (75.0)	126.21	12.90	96	149
Other education^∗^	28 (33.3)	6 (21.4)	22 (78.6)	175.00	18.73	145	212
Total	84 (100.0)	20 (23.8)	64 (76.2)	142.48	27.56	96	212

*^∗^Secondary or vocational education (younger than 18 years).*

### Procedure

All students had come for diagnostic assessment at the same university institute for learning difficulties. The child’s scores, as obtained from the diagnostic examination, were anonymously processed in an SPSS database. In order to diagnose DD, the following procedure was followed: (a) collection of information on (academic) performance from parents and school records (answers on standardized questionnaires and data from national mathematics tests); and (b) individual diagnostic examination (administered in two to three blocks of time, each lasting approximately 5 h). All diagnostic testing took place individually in a quiet space and was performed by a clinician with at least a master’s degree in psychology under supervision of a psychologist with a doctoral degree.

### Measures

The performance of the students on each primary factor was measured with one specific instrument (as part of the detailed procedure described in DDBP). A description of each instrument is provided below. Due to diagnostic considerations (age, time, the size of the test battery, etc.), not every instrument was used with every student. For each research measure, we indicated how many student scores were available. Seventy students (83.3%) were administered all measures. The instrument descriptions below include information on standardization (mean and standard deviation), and a cut-off score (mean minus one standard deviation) that indicates deficits in the specific area.

#### Planning

The Planning scale of the Cognitive Assessment System (CAS; individual test for children aged 5–17 years; Das and Naglieri, 1997) was used to measure *planning skills*. This scale consists of two (short version) or three (full version) subtests. In both cases a standardized score is derived (*M* = 100, *SD* = 15). The subtests are “Matching Numbers,” “Planned Codes,” and “Planned Connections” (Das and Naglieri, 1997). The “Matching Numbers” subtest consists of four pages, each with eight rows of six numbers. The numbers increase in size from one to seven digits. The student must underline the two corresponding numbers in each row. The “Planned Codes” subtest consists of two parts. A legend at the top of the page shows which codes belong to the letters A through D. The page contains 56 letters without codes arranged in different combinations. The student must fill in the correct code below each letter. The “Planned Connections” subtest consists of items that increase in difficulty. Each item consists of a page where numbers or letters, distributed randomly across the page, must be connected by the student in the correct order. The subtest scores are determined by both accuracy and speed (Das and Naglieri, 1997). A score below 85 indicates deficits in planning. The Planning scale was administered to 81 students (96.4%). The CAS has been found to provide a valid picture of information processing (Kroesbergen et al., 2002; Van Luit et al., 2005); the average reliability coefficient for the Planning scale is 0.88 (Das and Naglieri, 1997; Naglieri, 1999).

#### Naming Speed

Four cards of the Rapid Naming & Reading Test (RN&RT; Van den Bos and Lutje Spelberg, 2007) were used to measure the *naming speed* of colors, digits, pictures and letters. This task provides an indication of how quickly a student can extract verbal information about visual characters from memory. With each card, the student must identify one kind of visual character as quickly as possible. The time (in seconds) a student takes to name the characters on a single card is counted as the raw score; this is then converted to a standard score (*M* = 10, *SD* = 3). A standard score below seven indicates deficits in naming speed. The RN&RT was administered to 80 students (95.2%). Research has shown that the RN&RT is sufficiently reliable and valid (Van den Bos and Lutje Spelberg, 2007).

#### Short-Term and Working Memory

The computerized Automated Working Memory Assessment (AWMA; Alloway, 2007) was used to measure the capacity of *short-term and working memory*. Scores on the AWMA are fairly stable during the primary school period and show good convergence with WISC-IV memory tasks (Alloway et al., 2008). Each subtest out of six starts with a practice session and consists of blocks containing six trials. The first block of each trial consists of one stimulus. For each subsequent block, the trial increases by one stimulus. After three errors in one block, the task is terminated. After four correct answers in one block, the student can proceed to the next block and receives a maximum six points. In other cases, the score is the number of correct items per block. The raw score is the sum of all points scored. This raw score is converted to a standard score (*M* = 100, *SD* = 15) per subtest. The AWMA differentiates between four components of short-term and working memory. Verbal short-term memory is measured with the subtests “Digit recall,” “Word recall” and/or “Non-word recall.” In these three subtests the student hears a series of verbally presented numbers, words and/or nonsense words, and must then recall this series correctly. If more than one of the subtests was administered, an average for the verbal short-term memory was calculated based on information in the manual. Verbal working memory is measured with the subtest “Listening recall.” The student hears a series of spoken sentences, and at the end of each series must: (a) indicate whether the sentence is true or false, and (b) recall the last word of each sentence in sequence. (The true/false judgment is not included in the scores.) Visuospatial short-term memory is measured with the subtest “Dot matrix.” The student is shown the position of red dots in a matrix of 4 × 4 boxes for two seconds. The position of these dots must be identified in the correct order after the dots have disappeared. Visuospatial working memory is measured with the subtest “Odd one out”. The student views three shapes in boxes next to each other and identifies the shape different from the others. At the end of each trial, the student must identify, in the correct order, the location of each shape that was the odd-one-out. A standard score lower than 85 indicates deficits in memory. Deficits may occur on one specific memory component or multiple components at a time. The test–retest reliability for the subtests is, respectively, 0.84, 0.76, 0.64, 0.81, 0.83, and 0.81 (Alloway et al., 2006).

#### Attention

The Attention scale of the Cognitive Assessment System (CAS; individual test for children aged 5–17 years; Das and Naglieri, 1997) was used to measure *attention*. The scale consists of two (short version) or three (full version) subtests. In both cases a score was calculated (*M* = 100, *SD* = 15). The subtests are “Expressive Attention,” “Number Detection,” and “Receptive Attention” (Das and Naglieri, 1997). The “Expressive Attention” subtest consists of a page with words like “blue” and “red” printed in different colors. The student must name the color in which the words are printed; the dominant response, the word which is read, must be suppressed. Two exercises are taken in advance to determine whether the student is sufficiently capable of naming words and colors. The subtest score is determined based on speed and accuracy on the final task. The “Number Detection” subtest consists of two pages with numbers. These numbers are printed in different fonts. On each page the student must underline the numbers that look the same as those at the top of the page (e.g., 1, 2, 3 printed in open font). This requires selectively focusing attention on specifically printed numerical symbols. The “Receptive Attention” subtest consists of pictures or letters in pairs. The student must underline when the two pictures/letters are the same or have a similar characteristic. These subtest scores are also determined by accuracy and speed (Das and Naglieri, 1997). A scale score below 85 indicates deficits in attention. The Attention scale was administered to 79 students (94.0%). The CAS provides a valid picture of information processing (Kroesbergen et al., 2002; Van Luit et al., 2005), and the average reliability coefficient for the Attention scale is 0.88 (Das and Naglieri, 1997; Naglieri, 1999).

## Results

**Table 2** shows descriptive statistics for each factor and component. On nearly all measures the average participant scores were less than the mean scale or standard score (100 for planning, memory and attention, 10 for naming speed), though not lower than the criterion score indicating deficits in these factors (<85 for planning, memory and attention, <7 for naming speed). In **Table 3** correlations between all factors and components are presented. This table shows significant correlations between all factors (e.g., at least each factor correlated significantly with at least one other factor). A strong association (*r* > 0.50) was found between *planning skills* and *attention*, and between components within *naming speed*, i.e., colors-pictures, numbers-pictures, and letters-pictures). Moderate to strong associations (0.3 < *r* < 0.05) were found between *planning skills* and *naming speed* (i.e., colors); *naming speed* (i.e., colors) and *short-term/working memory* (i.e., visual STM); and *naming speed* (i.e., colors) and *attention.*

**Table 2:** Descriptive statistics for each factor and component.

Factor	Component	*n*	*M*	*SD*	Min	Max
**Planning**						
	Planning	81	86.47	12.77	57.00	127.00
**Naming speed**						
	Colors	80	8.58	3.39	1.00	16.00
	Numbers	80	8.36	3.36	2.00	16.00
	Pictures	80	8.68	3.30	1.00	16.00
	Letters	80	9.16	3.28	1.00	17.00
**Memory**						
	Verbal STM	81	96.07	13.37	68.00	132.00
	Verbal WM	82	109.72	14.31	74.00	138.00
	Visuospatial STM	80	98.75	18.53	62.00	141.00
	Visuospatial WM	79	96.92	14.54	65.00	139.00
**Attention**						
	Attention	79	95.81	11.65	63.00	133.00

*STM, short-term memory; WM, working memory.*

**Table 3:** Correlations between all factors and component.

Factor (– component)	1.	2.	3.	4.	5.	6.	7.	8.	9.	10.
(1) Planning	–	0.41^∗∗^	0.21	0.30^∗∗^	0.16	0.28^∗^	0.11	0.28^∗^	0.20	0.60^∗∗^
(2) Naming speed – colors		–	0.49^∗∗^	0.68^∗∗^	0.50^∗∗^	0.18	0.21	0.37^∗∗^	0.34^∗∗^	0.46^∗∗^
(3) Naming speed – numbers			–	0.55^∗∗^	0.66^∗∗^	0.01	−0.04	0.02	0.23^∗^	0.26^∗^
(4) Naming speed – pictures				–	0.60^∗∗^	0.30^∗∗^	0.23^∗^	0.33^∗∗^	0.41^∗∗^	0.36^∗^
(5) Naming speed – letters					–	0.14	0.04	0.16	0.22	0.25^∗^
(6) Memory – Verbal STM						–	0.32^∗∗^	0.09	0.32^∗∗^	0.27^∗^
(7) Memory – Verbal WM							–	0.21	0.40^∗∗^	0.13
(8) Memory – Visual STM								–	0.43^∗∗^	0.29^∗^
(9) Memory – Visual WM									–	0.33^∗∗^
(10) Attention										–

*^∗^ p < 0.05, ^∗∗^ p < 0.01, STM, short-term memory; WM, working memory.*

**Table 4** gives an overview of the number of students with deficits by factor and component. Deficits in *naming speed* were found in 54 students (64.3%), deficits in *short-term/working memory* in 41 students (49.4%), deficits in *planning skills* in 37 students (45.7%) and deficits in *attention* in 10 students (12.7%). Within the *naming speed* factor, deficits in naming numbers were the most common (*n* = 37, 46.3%) and deficits in naming colors were the least common (*n* = 26, 32.5%). Within the *short-term/working memory* factor, deficits in visual short-term memory were the most common (*n* = 21, 25.0%) and deficits in verbal working memory were the least common (*n* = 4, 4.9%). The number of students with deficits was associated with specific factors; there were significantly more students with deficits in *planning skills, naming speed* and *short-term/working memory* than in *attention* [χ^2^(9) = 69.63, *p* < 0.01].

**Table 4:** Numbers and percentages of students with deficits for each factor and component.

Factor	Component		Deficit		No deficit	
		*n*	*n*	%	*n*	%
Planning		81	37	45.7	44	54.3
Naming speed		80	54	67.5	26	32.5
	Colors	80	26	32.5	54	67.5
	Numbers	80	37	46.3	43	53.8
	Pictures	80	31	38.8	49	61.3
	Letters	80	27	33.8	53	66.3
Memory		83	41	49.4	42	50.6
	Verbal STM	81	15	18.5	66	81.5
	Verbal WM	82	4	4.9	78	95.1
	Visuospatial STM	80	21	26.3	59	73.8
	Visuospatial WM	79	16	20.3	63	79.7
Attention		79	10	12.7	69	87.3

*STM, short-term memory; WM, working memory.*

**Table 5** gives an overview of the distribution of deficits in the factors across students. To enhance clarity, in this table the components are integrated into information about the given factor. In other words, a deficit in one of the elements (subtests) comprising *naming speed* or *planning skills* has been considered as a deficit in that factor as a whole (instead of separate components within that factor. The first column shows the number of deficits (zero up to four) that could be present in students. The second column presents the number of students who experienced each deficit. In the remaining columns the contribution of the deficits over the four factors is shown. For example, **Table 5** shows that only one primary factor was found in 26 students (31.0%). Within these 26 pupils, 61.5% had deficits in *naming speed*, 23.1% had deficits in *short term and/or working memory* and 15.4% had deficits in *planning skills*. **Table 5** shows three findings. Firstly, in 13 of 84 pupils, no primary factor of DD was found. Secondly, the table shows that *naming speed* was the most common unique factor of mathematical problems, with 61.5% of students with a deficit in at least one component within naming speed. Thirdly, *attention* did not occur as a unique factor, but only in conjunction with at least two other primary factors of mathematical problems.

**Table 5:** Overview of the distribution of deficits in primary factors.

Number of deficits	Students with this number of deficits		Deficit in factor							
			Planning		Naming speed		Memory		Attention	
	*n*	%	*n*	%	*n*	%	*n*	%	*n*	%
0	13	15.5	0	0.0	0	0.0	0	0.0	0	0.0
1	26	31.0	4	15.4	16	61.5	6	23.1	0	0.0
2	25	29.8	14	56.0	19	76.0	17	68.0	0	0.0
3	14	16.7	13	92.9	13	92.9	12	85.7	4	28.6
4	6	7.1	6	100.0	6	100.0	6	100.0	6	100.0

## Discussion

The purpose of the current study was to describe the frequency of deficits on four primary associative factors for students with a diagnosis of DD: *planning, naming speed, short-term/working memory*, and *attention*. In 84 students aged 8–18 years with a diagnosis of DD (according to the DDBP protocol), the presence of deficits in these four factors was explored. Descriptive information showed that no primary factor of DD was found in 15.5% of the students. According to the DDBP protocol, establishing DD with certainty is difficult when the underlying factors of the mathematical problems remain unclear (Van Luit et al., 2014). The protocol indicates that, if no primary cause is found, a combination of secondary associative factors may also lead to the diagnosis. For those student participants with a diagnosis of DD but no underlying cognitive deficit, it may be that: (a) number sense served as an important factor when this factor is developed weak; (b) there were sufficient secondary factors that supported the diagnosis; (c) the student had above-average intelligence, meaning the criteria of deficits in *planning, naming speed, short-term/working memory* and/or *attention* were compensated for by other cognitive strengths; and/or (d) other primary associative factors, as yet not identified, played a role. Indeed, research has not yet been sufficiently conclusive to confirm that there are only five primary associative factors underlying DD (Van Luit et al., 2014; Träff et al., 2017).

The first research goal was to determine the percentage of students with deficits in the primary factors, indicating deficits in specific skills. The results show that deficits in naming speed (especially in naming numbers) were diagnosed most frequently, followed by deficits in short-term and working memory and in the field of planning. Deficits in attention were diagnosed least frequently. This could be explained in part by the fact that, in children with (probable) AD(H)D, research into DD typically does not take place before the symptoms of this disorder have been reduced due to therapy and/or medication.

The second research goal was to investigate the multifactorial distribution of deficits across these four primary factors in students with DD. Deficits on one or two associative factors were found for most students. In 15.5% of the students, no primary underlying factor for DD was found. In 31% of the students one primary underlying factor was found and in the remaining 53.6% of the children, deficits on three or four factors were found. A breakdown of the four factors provided two interesting insights into the presence of the four primary associative factors in students with DD. Firstly, naming speed was the most common unique factor of math problems. In more than half the cases (61.5%), students with DD had difficulty readily finding relevant information for solving a task. This finding is consistent with the results from studies of D’Amico and Passolunghi (2009), and Mazzocco and Grimm (2013). Secondly, in the current study, attentional deficits never appeared as a unique primary factor in pupils with DD, a finding also shown in previous research (e.g., Roeyers and Baeyens, 2016). Deficits in attention were only found when at least two other primary factors were present, and this was the case only for a small portion of the sample (11.9%). This means that focusing and sustaining attention can play a role in DD, but these abilities are not at the forefront for this particular clinical target group. As noted earlier, this finding is possibly due to the deferral of children diagnosed with (probable) AD(H)D. Deficits in *naming speed, short-term/working memory* and *planning* were found to be the factors occurring most frequently in the student participants.

Although this research was exploratory and descriptive, by differentiating among cognitive deficits as may underly DD, our findings nevertheless support the line of research focusing on the heterogeneity of this clinical condition (e.g., Skagerlund and Träff, 2016). The findings also highlight the added value of systematically investigating primary factors during individual diagnostic research, as encouraged in the DDBP protocol. The results therefore emphasize the need for behavioral experts to investigate these factors as extensively as possible when conducting diagnostic research in severe mathematical problems. As stated in the DDBP protocol (Van Luit et al., 2014), having insight into a student’s performance in these (primary) underlying factors can help experts address their problems, by giving them additional understanding of the specific educational needs of the student.

An important limitation of the current research is the absence of analysis into number sense. Research has shown that the ability to process, understand and estimate numerical quantities (Dehaene, 1992) is a predictive factor in the development of mathematical skills (Fuchs et al., 2010; LeFevre et al., 2010; De Smedt and Gilmore, 2011). Unfortunately, as noted, the lack of valid standardized tests at the start of our data collection led to the omission of this factor in this study. There have been some promising relevant testing protocols (e.g., Jordan et al., 2004), but until this moment such tests have not been standardized. In follow up research, it may be possible to investigate if deficits in this area comprise an important primary factor for DD; however, this must await a reliable and valid number sense test. Follow up research also could systematically explore the effects of secondary factors within the physical, social and educational environment. Issues such as motivation, working attitude, competence-perception and/or performance anxiety in individual students may also exert a sizable influence on performance in mathematics. These were not considered in our study.

Another limitation of the current investigation is the exclusive focus on a clinical sample. It would be desirable for follow up research to compare an atypical sample of students with a control group of students without DD. This would allow the observed frequency of primary factors in students with DD to be compared with the occurrence of deficits in planning, naming speed, memory and attention within the normal population. Furthermore, in the current study, the clinical group originates from the client population of a single institution, which introduces the possibility of specificity. It would be useful for further research to gather broader information in order to form a more precise picture of the presence of primary and secondary factors in a more generalisable sample than in this current investigation. Nevertheless, the exploratory and descriptive nature of current research provides useful (clinical) information on systematic investigation of primary factors in students with (probable) DD.

## Author Contributions

JVL wrote the protocol for this research. ST analyzed the data. JVL and ST wrote the text.

## Conflict of Interest Statement

The authors declare that the research was conducted in the absence of any commercial or financial relationships that could be construed as a potential conflict of interest.

## Footnotes

1 The fifth factor, number sense, was not included in this research because of the time frame of the study (in the DDBP protocol, number sense has only been considered a primary factor since 2014; Van Luit et al., 2014). This meant that, first, number sense skills had been measured in too few children and, second, too much variation between test materials made comparison of the available data on number sense impossible.
